# Are network growth and the contributions to congresses associated with publication success? A pediatric oncology model

**DOI:** 10.1371/journal.pone.0210994

**Published:** 2019-01-25

**Authors:** Frank Berthold, Christoph Bartenhagen, Lothar Krempel

**Affiliations:** 1 University of Cologne, Department of Pediatric Oncology and Hematology, University of Cologne, Koeln, Germany; 2 Max Planck Institute for the Study of Societies, Koeln, Germany; KU Leuven, BELGIUM

## Abstract

**Background:**

The consistent focus of ‘Advances in Neuroblastoma Research’ congresses on the topic neuroblastoma sets it as a model for a circumscribed scientific community.

**Methods:**

The contributions of authors, institutions and countries to congress abstracts and their collaborations were compared to the Hirsch index (h*-*index) calculated from the Web of Science publication output on the topic ‘neuroblastoma’.

**Results:**

From 1975 to 2016, 18 congresses were held. 8459 authors affiliated to 553 institutions of 53 countries presented 3,993 abstracts. The number of coauthors increased over the years from 2 to 7. A considerable proportion of authors, institutions and countries presented only once (53.7%/25.7%/13.2%). Authors with a high number of abstracts and with a large local network were often among those with a higher publication rate and success (R^2^ = 0.508 for Pearson’s correlation between weight and h-index, R^2^ = 0.474 for degree centrality, R^2^ = 0.364 for lobby-index). Closeness and betweenness centralities were less correlated (R^2^ = 0.127/R^2^ = 0.33, resp.). The institutions showed a similar impact of local interactions on publication success (degree centrality R^2^ = 0.417, weight R^2^ = 0.308), while countries demonstrated a higher correlation of betweenness centrality and h-Index (R^2^ = 0.704) emphasizing their brokerage role. Of 553 institutions, 520 collaborated within 13 communities and belonged to the large scientific network. 33 satellite institutions had no connections to the central network. They attended 1–4 congresses over a period of 1–16 years.

**Conclusion:**

A large scientific network has been developed during the recent 42 years. Growth and interaction at congresses were correlated to publication success. Weight is suggested as a useful and simple estimate.

## Introduction

Neuroblastoma is a malignant tumor of the peripheral sympathetic nervous system and represents the most frequent solid tumor in childhood [1]. Congresses on ‘Advances in Neuroblastoma Research’ (ANR) convened experimental scientists and paediatric oncologists from all-over the world since 1975. The ANR congresses were founded in Philadelphia, USA, by Dr. Audrey Evans and rotated after 1990 all over the continents. The exclusive focus on one disease from different points of views renders the meetings to a model for the development of a circumscribed scientific network.

The use of congresses for the development of a scientific community and for the output of articles is a matter of debate. The collaborations of authors, institutions and the related countries can be evaluated by the analysis of co-authorships on congress abstracts. International co-authorship in scientific papers is considered as proxy for international collaboration [2]. Publication success is measured by the number of publications (scientific output) and the number of citations (scientific impact). Both can be estimated by the specified Hirsch index (h-index) [3, 4]. This study describes the development of a scientific community over a long period of time and asks the question of the best parameter for predicting later scientific success. The methods used are applicable to any series of congresses with a theme that is confined to one specific topic, i.e. in medicine to one disease.

## Material and methods

The published abstracts of the 18 ANR congresses held between 1975 and 2016 were investigated regarding authors, affiliated institutions and the countries of the institutions.

### Abstracts

Abstracts were submitted to the local congress organization, independently evaluated and published in printed form (congress books). The number of rejected abstracts is unknown. Abstracts of workshops were mainly invited and not considered in this analysis. The congress organizers categorized into one of the three categories (basic, translational, clinical science) according to their main content.

### Authors

Each author was present at least once as a (co-)author at a congress abstract. Different spellings of one author were modified into one.

### Institutions

The institutions of the authors were named according to the city of location. Different sub-specialties (e.g. radiology, pathology, pediatric oncology, genetics) were not discriminated and counted as one institution. Three exemptions were made when two or more large institutions were located in one city (Paris 1 (Institute Curie), 2 (Villejuif), 3 (other), New York 1 (MSKCC), 2 (Fordham, Bronx), 3 (other), Moscow 1 (Rogatschew), 2 (Plochin).

### Countries

The location of an institution determined the country. In case of change of the name of the country (e.g. Yugoslavia to Serbia, Croatia), the designation of the year 2017 was chosen.

Several measures and data structures were applied to investigate the scientific network, collaboration and influence of authors, institutions and countries within the community as well as their scientific output and success.

### Co-authorship network

Interactions within the scientific network were measured using three undirected, unweighted graphs, where the nodes represent the authors, institutions or countries and the edges represent a joint contribution to one or more abstracts.

### Centralities

Based on the graph, degree (local connections), closeness (global connections), betweenness (brokerage), and Eigenvector (leadership) centralities were calculated from the presence of authors, institutions, and countries at ANR congresses derived from authorships and affiliations.

### Communities

The Louvain method [5] was used to identify communities within the largest scientific network by modularity optimization. A community is a tightly knit group characterized by a higher density of ties compared to all other connections. Each institution can belong only to one community.

### Satellite institutions

Satellite groups were defined by the lack of any connection to the largest scientific network.

### Weight

The weight of an author, institution or country was the sum of the weights of the corresponding abstracts. Every abstract had a weight of one. Of multi-authored abstracts, the weight was adjusted by fractionalized counting [6], e.g. in case of 10 authors each abstract had a weight of 0.1. The fractionalized counting method was likewise applied to affiliated institutions and countries.

### Publications

The publications were downloaded from the Web of Science for the time span 1975–2017 using published articles with the topic ‘neuroblastoma’. The search covered headlines, abstracts, and key words of published articles. The search according to the topic was preferred over the title alone [7]. Abstracts, meeting reports, and reviews were excluded. Access date was July 17^th^, 2017.

### Hirsch index

Mathematically, the Hirsch index (h-index) can be defined as an operator on a set of integers X = (x_1_,…,x_n_), which returns the maximum integer y>0, such that there are at least y elements in X, each being greater than or equal to h [8]. In [3], the h-index was first introduced as a performance measure to characterize a researchers output. It is defined as the maximum number h of publications with at least h citations. To compute the h-index of institutions and countries, the number of publications and citations of all their associated authors were added up.

### Molinari index

The h-index is expected to grow with the number of publications, which introduces a size bias for large institutions and countries. To correct this size dependency, Molinari et al. (9) described the h_m_-index, a modification of the h-index: h_m_ = h/n^b^, where n^b^ is the growth rate for n publications and the slope b of the growth curve at 200 publications. Our parameter b = 0.42 is consistent with the observation in [9], that the slope is usually the same (~0.4) across many large sets of publications (e.g. by institution or journal). However, this does not hold true when the number of publications is too small (e.g. below 100). Hence, the h_m_-index only becomes meaningful and comparable for larger sets.

### Citation rate

As another metric to measure scientific impact in large sets of publications, we calculated the citation rate for each institution and country, i.e. the average number of citations, by dividing the total number of citations received by all publications of an institution/country by its total number of publications. This was done for all institutions and countries with 50 or more publications.

### Lobby index

The definition of the h-index can be applied to other data structures such as graphs [8, 10, 11]. In [12], Korn et al. introduced the lobby index (l-index) as a centrality measure. It is defined for each node as the maximum number l of neighbors with a degree of at least l. The l-index captures the efficiency of interaction within the network [12].

### Software

Basic statistics (e.g. counting, correlation analyses, plots) were performed using the Excel 2010 program for Windows. Publication and citation statistics were from the Web of Science using the web interface and its search and export utilities. The lists were further processed and mean citation-, h- l-, and h_m_-indices were calculated with R version 3.3.3. Centralities and communities were calculated using the PAJEK 5.2 program (GEPHI version 9.2). The IBM SSPS statistical package version 25 was used for multivariable analysis.

## Results

### 1. Development of the scientific network at ANR congresses

#### 1.1. The contributions of authors, institutions, and countries to the congress abstracts

From 1975 through 2016, 18 congresses were held and 3,993 abstracts published. The number of abstracts per congress increased from 41 in 1975 to a maximum of 458 in 2014. 8,459 authors (range per congress 41–1,899) from 553 institutions (range 13–236) of 53 countries (range 4–36) contributed.

**Fig 1:**
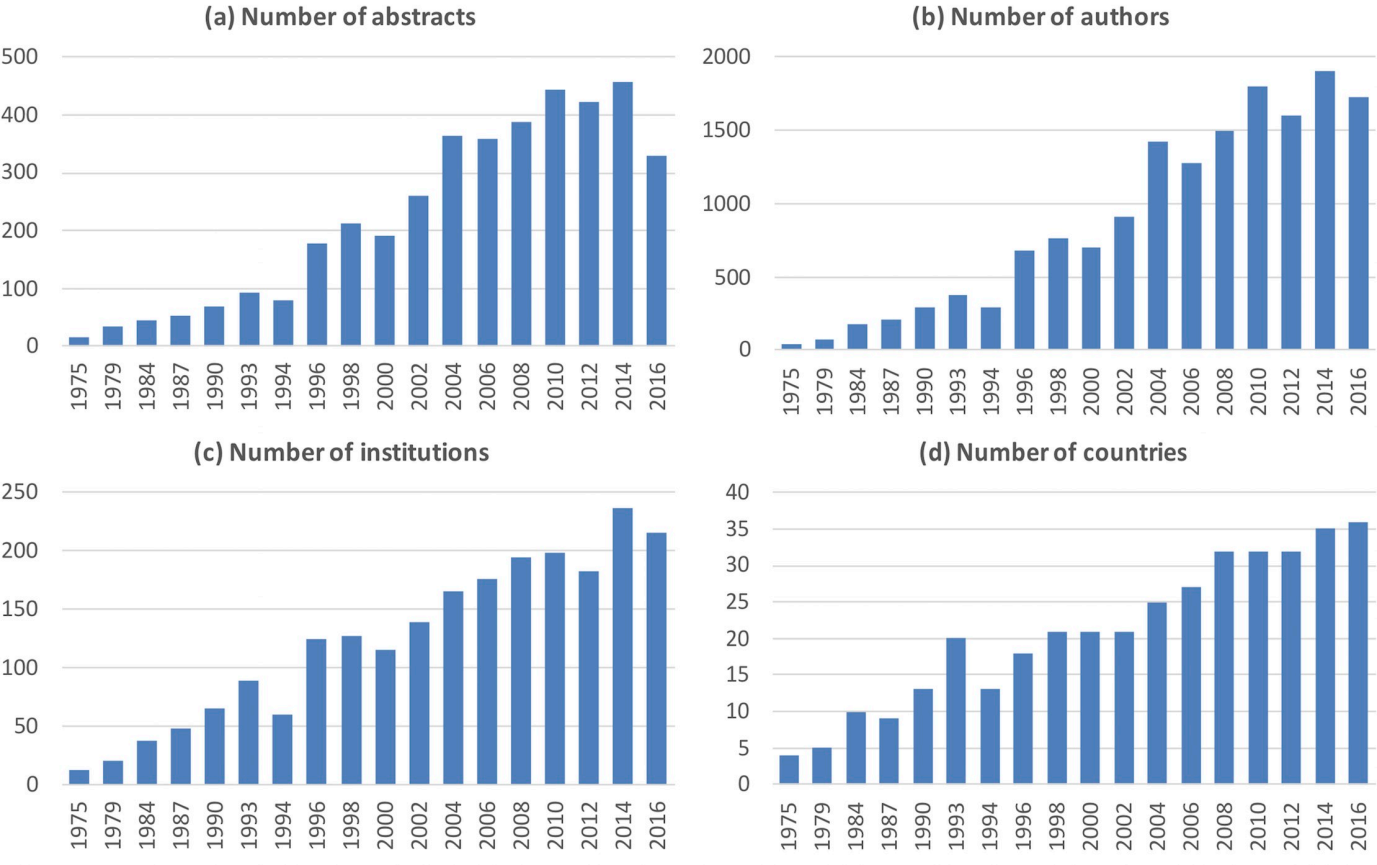
The numbers of (a) abstracts, (b) authors, (c) institutions and (d) countries per year.

One country (USA) was 18x present, 4 countries 17x, 2 countries 16x, 7 countries 2x, and 12 countries only once. “Fig 1” shows an almost linear increase of abstracts, authors, institutions, and countries until 2010 and a saturation effect thereafter (abstracts 328–458, authors 1,722–1,804, institutions 199–236, countries 32–36).

**Fig 2:**
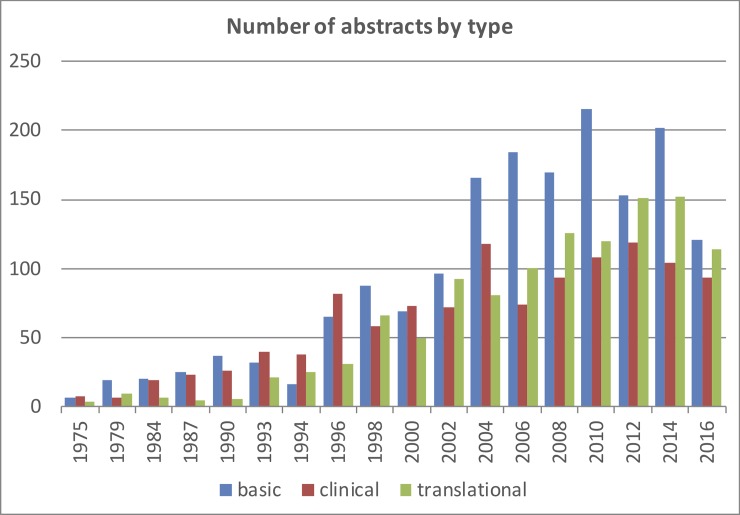
The proportions of abstract types per year.

The abstract types belonged in 42.2% to basic, in 28.9% each to translational and clinical science. Basic and translational contributions were in parallel between 1975 and 2002. After 2004 basic science was prominent. Clinical abstracts were minimal until 1993, increased in the following years and almost in equal proportions as translational abstracts between 1998 and 2010 (“Fig 2”).

The mean number of co-authors per abstract was 5.2 increasing from 2.3 in 1975 to 7.4 in 2016.

The total count of names (every abstract, every name of author counted) was 28,640.

#### 1.2 The impact of selected subgroups of authors, institutions, and countries to abstract production

Of 8,459 authors, 4,860 contributed only to one abstract (57.5%) and 6,230 (73.6%) to 1 or 2 abstracts. The top 20 authors (co-)authored 101–171 abstracts.

Of 553 institutions, 143 (25.9%) had one and 214 (38.7%) two abstracts at the ANR congresses. The top 20 institutions were listed 393–1,655 times as contributors (more than count per abstract possible if >1 author of the same institution contributed to the same abstract).

Of 53 countries, seven (13.2%) had one and eight (15.1%) two abstracts in the ANR abstract books. The top 20 countries were listed 141–7,645 times, the top 10 1,077–7,645 times (24,534/28,640 = 85.7% of counts).

**Fig 3:**
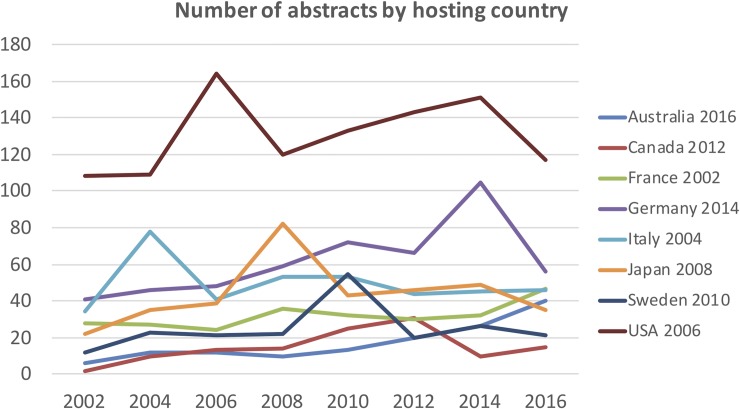
The influence of hosting an ANR congress on the number of abstracts.

Hosting an ANR congress stimulated the presence of the hosting country in that year and decreased thereafter back to the pre-meeting level (“Fig 3”).

### 2. Geographical representation

USA/Canada, Europe, Japan, and Australia are major areas of neuroblastoma research, while South America and Africa were hardly involved. A more detailed map of the US shows one large and one smaller community within the ANR network. Philadelphia, the founder institution and host of many congresses in particular during the first 20 years, is prominent in respect to the number of connections. Los Angeles, San Francisco, and New York were next. In Europe five major communities were identified. The largest community represents an internationally collaborating group (SIOPEN) with partners in all European countries with active neuroblastoma research. Four other communities appear to be mainly characterized by language (German, Italian, Polish, Spanish), although all partners are connected with institutions of different mother tongues.

### 3. Satellite groups

**Fig 4:**
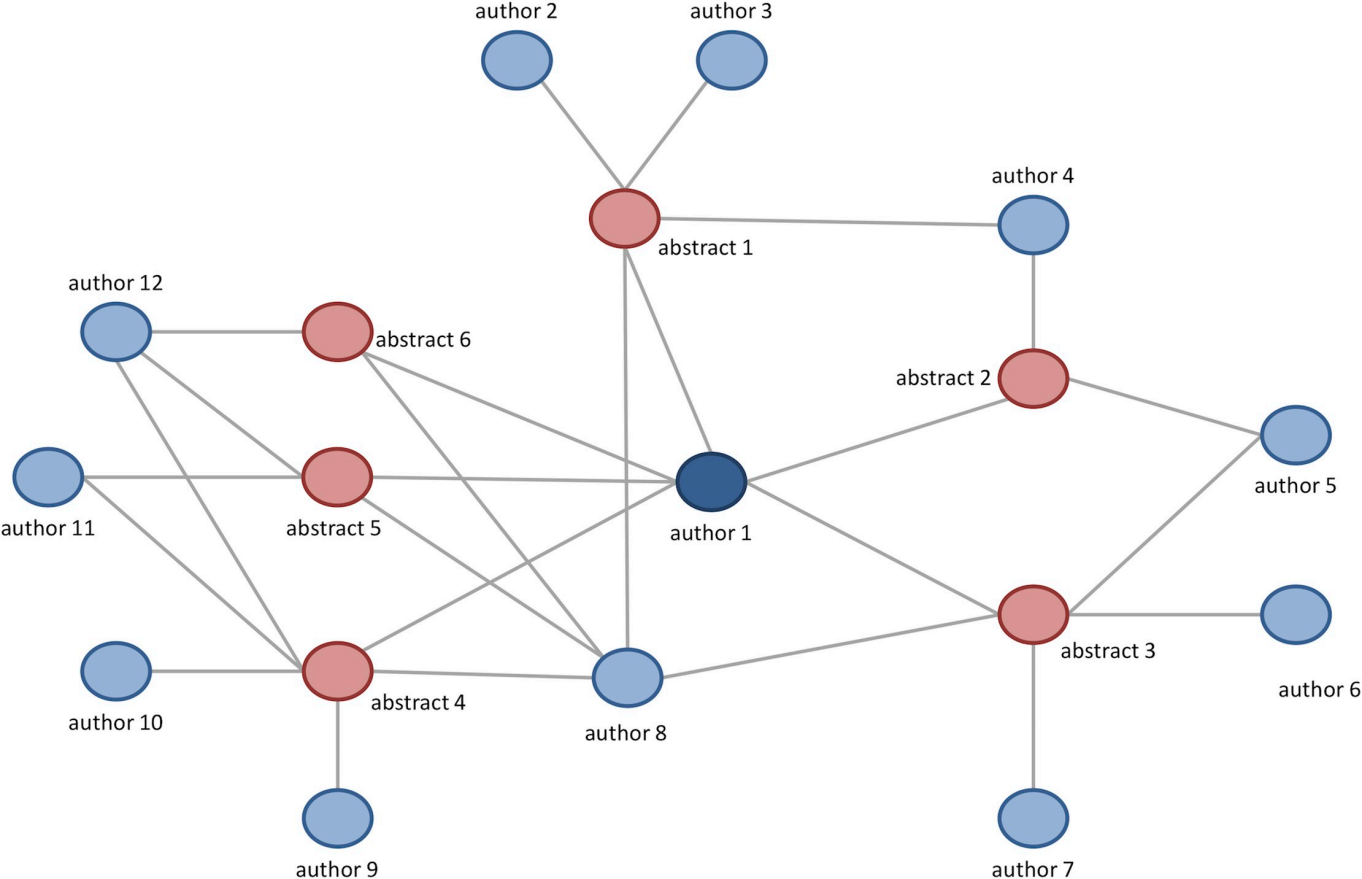
Example of a satellite group.

The large central network consisted of 8,235 authors and 520 institutions from 42 countries. Satellite groups without a single link to the central scientific network at any time point during their participation in ANR congresses had 224 authors from 33 institutions of 22 countries. Ten countries had satellite institutions only. Twelve countries had as well satellite institutions as institutions connected with the large network. The satellite institutions attended one to four ANR congresses over a time span of one to sixteen years. An example of a satellite group lasting 4 years (2010–2014) is shown in “Fig 4”.

### 4. The correlation of authors’ networking and publications

The 8,459 authors who participated in at least one ANR congress, contributed to 0–202 published articles per author under the topic neuroblastoma. The citations of the authors ranged between 0–12,930. The average citations per article ranged between 0–8,825. The h-index of all authors had a range of 0–61.

**Fig 5:**
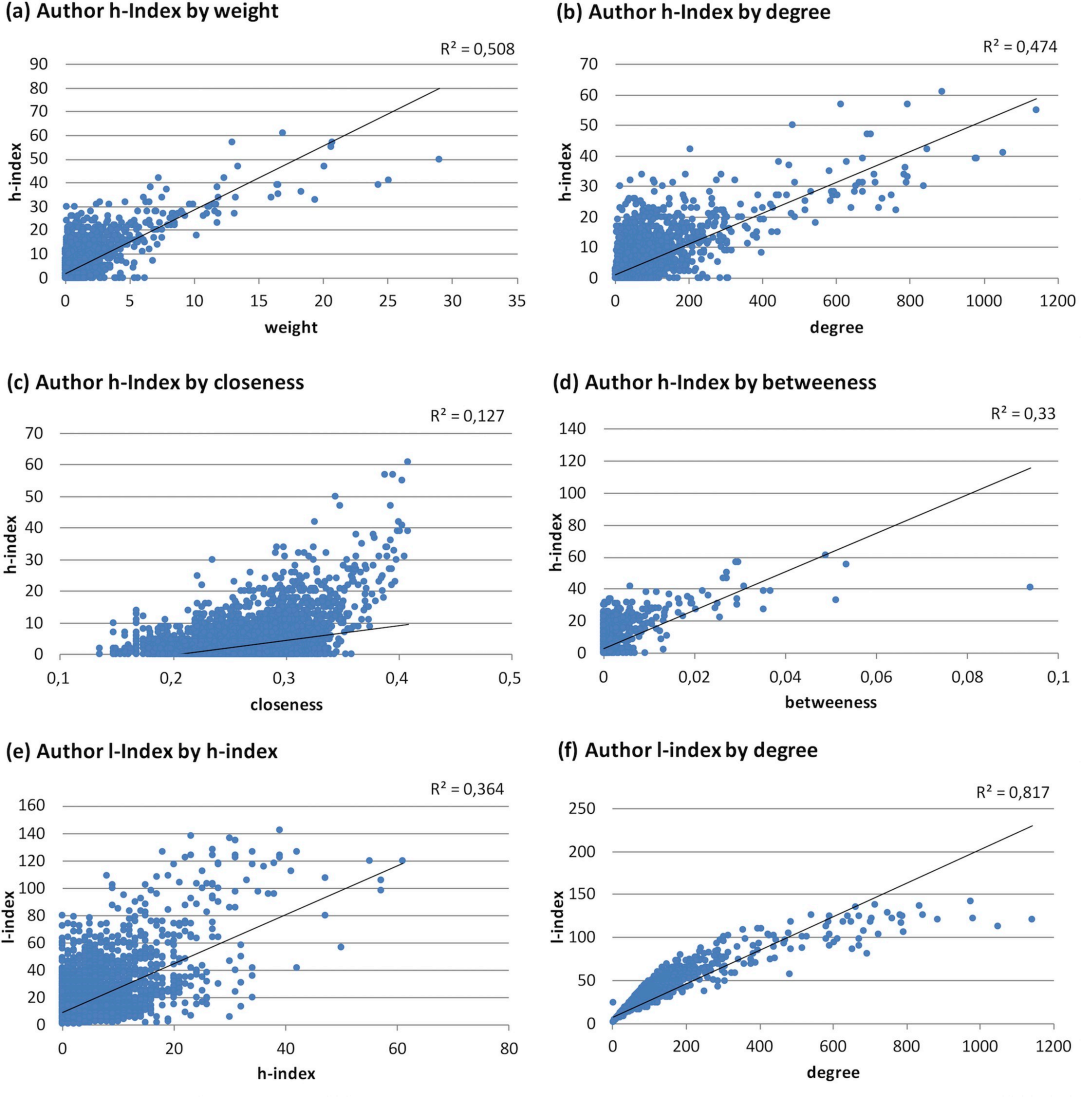
The correlation of h-index of neuroblastoma papers and author’s networking at ANR congresses estimated by (a) weight, (b) degree, (c) closeness, (d) betweenness and (e) l-index. In (f), the l-index is compared to the degree. The global centralities closeness and betweenness were only considered for the large central network of 8,235 authors.

“Fig 5” shows the correlations between author’s networking activities at ANR congresses and h*-*index of consecutive neuroblastoma papers. High presence on ANR congresses was associated with high h-indices. An h-index up to 30 could be achieved as single authors or as members of satellites with closeness and betweenness values of 0. Authors with an h*-*index >40 usually had 200 and more co-authors (up to nearly 1,000). This is reflected in the correlation between h-index and degree (R^2^ = 0.474, “Fig 5B”) and the l-index (R^2^ = 0.364, “Fig 5E”). However, the weight of contributions to abstracts had a higher correlation to the publication success (R^2^ = 0.508, “Fig 5A”) compared to betweenness (R^2^ = 0.33, “Fig 5D”) and closeness (R^2^ = 0.127, “Fig 5C”). In a multivariable model weight and all centralities showed an independent impact predicting the h*-*index (Table 1).

**Table 1:** Multivariable regression analysis of congress networking (independent variables) on publication success (dependent variables).

dependent variable	independent variable	regression coefficient B	95% confidence interval	logrank p-value	R^2^
h*-*index authors	weight	2.179	2.062; 2.295	**<0.001**	**0.549**
	degree	0.014	0.009; 0.019	**<0.001**	
	closeness	2.434	1.351; 3.518	**<0.001**	
	betweenness	-279.151	-348.802; -209.496	**<0.001**	
	l-index	0.041	0.025; 0.058	**<0.001**	
h*-*index institutions	weight	0.239	0.131; 0.347	**<0.001**	**0.451**
	degree	0.202	0.157; 0.247	**<0.001**	
	closeness	15.307	1.791; 28.823	**0.027**	
	betweenness	-470.080	-752.954; -187.205	**0.001**	
h_m_*-*index institutions	weight	0.010	0.000; 0.021	0.058	**0.218**
	degree	0.014	0.009; 0.018	**<0.001**	
	closeness	2.181	0.865; 3.497	**0.001**	
	betweenness	-50.673	-78.226; -23.121	**<0.001**	
h*-*index countries	weight	0.127	0.085; 0.169	**<0.001**	**0.862**
	degree	0.413	0.040; 0.786	**0.031**	
	closeness	19.224	-17.190; 55.639	0.294	
	betweenness	254.251	-241.709; 750.210	0.308	
h_m_*-*index countries	weight	-0.001	-0.003; 0.001	0.582	**0.442**
	degree	0.026	0.007; 0.044	**0.007**	
	closeness	0.476	-1.300; 2.252	0.592	
	betweenness	-1.533	-25.721; 22.655	0.899	
h*-*index communities	mean weight	14.050	0.497; 27.602	**0.044**	**0.881**
	mean degree	-0.449	-3.453; 2.555	0.739	
	mean closeness	832.122	-139.780; 1,804.023	0.084	
	mean betweenness	-3,070.989	-34,528.489; 28,386.511	0.828	
h_m_*-*index communities	mean weight	0.127	-0.122; 0.377	0.273	**0.722**
	mean degree	-0.062	-0.117; -0.006	**0.034**	
	mean closeness	26.804	8.886; 44.722	**0.009**	
	mean betweenness	-282.297	-862.250; 297.656	0.294	

The multivariable model includes the networking centralities to infer publication success estimated by h- or h_m_-index for authors, institutions, countries and communities of institutions. Their effect in the model is given by the regression coefficient B (positive effect for B>0, negative effect for B<0). Significant logrank test results with p<0.05 are depicted in bold.

Ranked by h*-*index (Table 2), the top 20 authors (0.24% of all) had an h*-*index between 34 and 61, 91–202 publications (total range 0–202), and 3,492–12,930 citations (total range 0–12,930). The weights of the top 20 authors were in the range of 6.642 and 29.0 (total range 0.026–29.0). The degree centrality of the top 20 authors ranged between 204 and 1,142 (total range 2–1,142), the closeness centrality between 0.344 and 0.409 (total range 0–0.409), and the betweenness centrality between 0.006 and 0.094 (total range 0–0.094).

**Table 2:** Comparison of top 20 h-index authors with ranks of abstract weights and centrality measures.

author	h*-*index	publi-cations	citations	weight rank	degree rank	l-index rank	closeness rank	betweenness rank	rank sum	total rank
A 01	61	128	11,296	9	5	16	1	4	35	4
A 02	57	154	10,228	17	8	30	22	9	86	10
A 03	57	148	12,930	4	29	42	12	12	99	12
A 04	55	202	9,956	5	1	17	5	2	30	1
A 05	50	202	6,569	1	43	172	110	13	339	19
A 06	47	143	6,658	14	20	29	16	14	93	11
A 07	47	135	8,301	6	19	69	96	15	205	17
A 08	42	99	6,007	18	6	6	7	8	45	6
A 09	42	99	4,342	53	150	317	256	87	863	20
A 10	41	175	6,444	2	2	24	4	1	33	3
A 11	39	171	6,470	3	3	13	6	5	30	2
A 12	39	122	5,390	10	4	1	8	20	43	5
A 13	39	127	6,367	12	21	9	2	6	50	7
A 14	38	93	3,922	62	28	18	32	42	182	15
A 15	38	98	4,671	21	47	46	53	55	222	18
A 16	37	91	6,564	47	45	47	30	30	199	16
A 17	36	119	4,206	8	11	23	15	19	76	8
A 18	35	112	4,389	11	36	44	48	25	164	14
A 19	34	95	3,492	20	12	7	21	24	84	9
A 20	34	93	3,915	49	18	20	19	10	116	13

The table shows the 20 most successful authors according to h-index and compares their ranks according to weight, degree, closeness, betweenness and l-index among all authors. A total rank of each author among the top 20 is given by the ranking of the sum of all five ranks.

The names of the top 20 authors are all well-known in the neuroblastoma science community. The authors with high h-indices had top ranks in the weight and centrality tables. The lowest rank of a top 20 author was 317 in the list of 8,459, 17 authors were also ranked in the top 20 of at least one centrality measure. Table 2 demonstrates that the ranks of the various measures were surprisingly close to each other. The lowest total rank authors had frequently low ranks in all of the five specific ranking lists.

### 5. The correlation of institution’s networking and publications

The 553 institutions participated in at least one ANR congress. Institutions contributed to 0–987 published articles under the topic neuroblastoma (top 20: 254–854). The citations of the institutions ranged between 0 and 37,373 (top20: 16,362–37,373). The h-index of all institutions had a range of 0–96 and the h_m_-index of 0–6.85.

The h-index of institutions showed good correlations with the degree centrality of institutions (R^2^ = 0.417) and the weight (R^2^ = 0.308), but less with the betweenness centrality (R^2^ = 0.268) and with the closeness centrality (R^2^ = 0.193). All centralities were significantly associated with the h-index by multivariable analysis (Table 1).

Larger institutions tend to have higher h-indices (R^2^ = 0.397). Therefore, we also compared the h_m_-index. However, it was poorly correlated the chosen networking measures: The R^2^ values were 0.086 for weight, 0.176 for degree, 0.136 for closeness and 0.07 for betweenness. The multivariable analysis for the h_m_-index selected all centralities but excluded weight (Table 1).

The general correlation between h- and h_m-_index was good (R^2^ = 0.699), but this was less evident for the top 20 institutions. Nine institutions (of 20) were present in both lists. However, the names of most of the top 20 institutions in the h_m_-index list are rather unknown in the neuroblastoma science community.

Of the 553 institutions, the lowest rank of a top 20 was 339 in the h-index list (Table 3) and 489 in the h_m_-index list (Table 4). All of the top 20 institutions published 254 or more papers. The minimum number of papers of the top 20 h_m_-index institutions was 44 only.

**Table 3:** Comparison of top 20 *h-*index institutions with ranks of abstract weights and centrality measures.

insti tution	h-index	publi-cations	citations	weight rank	degree rank	closeness rank	betweenness rank	rank sum	total rank
I 01	96	854	37,373	1	1	1	2	5	1
I 02	88	748	32,590	11	15	16	9	51	4
I 03	87	649	31,892	86	96	111	61	354	18
I 04	86	720	34,336	30	13	11	21	75	7
I 05	85	578	28,390	4	5	5	8	22	2
I 06	85	424	29,041	25	11	12	12	60	5
I 07	76	987	28,894	15	17	23	6	61	6
I 08	75	317	22,403	66	70	79	48	263	16
I 09	75	773	26,736	23	7	7	13	50	3
I 10	75	507	23,940	32	104	100	54	290	17
I 11	73	433	18,939	3	44	46	35	128	12
I 12	70	525	20,775	19	20	18	25	82	9
I 13	70	474	20,374	20	36	47	32	135	13
I 14	69	370	17,094	114	117	91	138	460	19
I 15	69	439	18,232	27	28	33	34	122	11
I 16	69	254	17,943	91	50	71	33	245	15
I 17	68	455	17,966	9	30	24	16	79	8
I 18	68	467	17,797	336	339	262	263	1,200	20
I 19	67	374	16,362	45	45	64	57	211	14
I 20	67	800	22,359	16	29	30	24	99	10

The table shows the 20 most successful institutions according to h-index and compares their ranks according to weight, degree, closeness and betweenness among all institutions. A total rank of each institution among the top 20 is given by the ranking of the sum of all four ranks.

**Table 4:** Comparison of top 20 h_m_-index institutions with ranks of abstract weights and centrality measures.

insti- tution	h_m_-index	publi- cations	citations	weight rank	degree rank	closeness rank	betweenness rank	rank sum	total rank
I 01	6.85	104	7,917	182	136	158	175	651	17
I 02	6.77	254	17,943	91	50	71	33	245	9
I 03	6.73	424	29,041	25	11	12	12	60	3
I 04	6.7	317	22,403	66	70	79	48	263	10
I 05	6.24	239	12,199	62	15	15	29	121	4
I 06	6.15	220	12,172	194	40	37	88	359	12
I 07	6.14	44	1,793	290	357	489	283	1,419	19
I 08	5.91	578	28,390	4	5	5	8	22	2
I 09	5.91	272	15,188	118	85	122	95	420	13
I 10	5.81	242	12,365	41	33	27	40	141	6
I 11	5.79	205	12,656	115	153	131	162	561	16
I 12	5.78	370	17,094	114	117	91	138	460	14
I 13	5.76	649	31,892	86	96	111	61	354	11
I 14	5.73	433	18,939	3	44	46	35	128	5
I 15	5.68	139	6,781	465	469	458	284	1,676	20
I 16	5.66	854	37,373	1	1	1	2	5	1
I 17	5.59	374	16,362	45	45	64	57	211	7
I 18	5.59	322	15,160	124	180	105	142	551	15
I 19	5.54	209	10,740	75	48	54	59	236	8
I 20	5.53	87	3,692	358	312	251	152	1,073	18

In comparison to Table 4, this table shows the institution rankings according to the h_m_-index. Nine institutions appear in both h- and h_m_-index top 20 lists, although with different ranking.

As another measure for scientific impact, we looked at the citation rate of institutions. It was highly correlated to the h_m_-index (R^2^ = 0.781). However, correlation to h-index (R^2^ = 0.367) and weight, degree, closeness and betweenness (all R^2^ below 0.1) was lower compared to the h_m_-index. For h- and h_m_-index 12 and 9 of the top 20 institutions ranked also among the top 20 of at least one of the centrality measures.

### 6. The correlation of countries’ networking and publications

The authors of 53 countries presented in at least one ANR congress. Those authors published consecutively to 0–9,899 articles under the topic neuroblastoma. The citations ranged between 790 and 353,122. The h-index of all countries had a range of 12–198 and the h_m_-index of 1.81–3.88.

The correlation of the h-index of countries with the weight of their authors on ANR abstracts (R^2^ = 0.748) and the betweenness (R^2^ = 0.704) was very high. The degree (R^2^ = 0.481) and the closeness (R^2^ = 0.365) were less correlated. By multivariable analysis only weight and degree were independently predictive of the h-index of countries (Table 1).

The number of researchers in a country was highly correlated to the h-index (R^2^ = 0.753). The size independent h_m_-index was moderately correlated to the degree and closeness centralities (R^2^ = 0.424 and R^2^ = 0.342 respectively) and poorly associated with betweenness centrality (R^2^ = 0.103) and weight (R^2^ = 0.039). The multivariable model selected only degree centrality as independently predictive for the h_m_-index of countries (Table 1).

The top 10 countries ranked by the h-index were all well-known as very active in neuroblastoma research, while the top 10 counties sorted by the h_m_-index were mainly small countries and considered as less prominent by the neuroblastoma science community. The countries with high h-indices had comparable top ranks in the weight and centrality tables (Table 5). Table 6 shows ranks between 5 and 29 (of 53 countries) for the top 10 ranked by the h_m_-index indicating no top ranks (1–4) and a wide variability of ranks within the applied measures.

**Table 5:** Comparison of top 10 h*-*index countries with ranks of abstract weights and centrality measures.

country	h-index	publi-cations	citations	weight rank	degree rank	closeness rank	betweenness rank	rank sum	total rank
C 01	198	9,899	353,122	1	6	5	1	13	2
C 02	114	2,676	82,093	2	1	1	3	7	1
C 03	113	3,163	86,431	3	22	19	4	48	8
C 04	106	2,633	70,022	4	2	2	10	18	4
C 05	102	1,931	58,210	6	3	4	6	19	5
C 06	92	2,531	54,828	5	7	6	5	23	6
C 07	85	1,135	34,508	13	23	21	17	74	10
C 08	73	614	21,544	8	17	15	12	52	9
C 09	71	1,065	26,910	12	10	10	7	39	7
C 10	69	844	24,432	7	4	3	2	16	3

**Table 6:** Comparison of top 10 h_m_-index countries with ranks of abstract weights and centrality measures.

country	h_m_-index	publi- cations	citations	weight rank	degree rank	closeness rank	betweenness rank	rank sum	total rank
C 01	3.88	156	7,019	21	12	11	18	62	5
C 02	3.81	190	6,214	20	13	12	16	61	6
C 03	3.8	482	16,940	18	14	15	20	67	4
C 04	3.76	614	21,544	8	17	16	12	53	7
C 05	3.66	543	18,669	14	10	13	13	50	8
C 06	3.51	466	14,731	9	5	5	9	28	10
C 07	3.45	370	11,443	11	6	6	15	38	9
C 08	3.37	149	4,659	28	26	26	8	88	1
C 09	3.33	143	4,434	29	15	14	19	77	2
C 10	3.3	1,135	34,508	13	22	21	17	73	3

Similar to institutions, the correlation between the citation rate and h_m_-index was high (R^2^ = 0.787), but relatively low for h-index (R^2^ = 0.279), betweenness (R^2^ = 0.157) and weight (R^2^ = 0.131). Degree and closeness on the other side, were moderately associated with R^2^ = 0.453 and R^2^ = 0.334 respectively.

The table shows the 10 most successful countries according to h-index and compares their ranks according to weight, degree, closeness and betweenness among all countries. A total rank of each country among the top 10 is given by the ranking of the sum of all four ranks.

In comparison to Table 4, this table shows country rankings according to the h_m_-index. Two countries appear in both h- and h_m_-index top 20 lists, although with different ranking.

### 7. Communities

The Louvain algorithm identified 13 communities within the network of 520 institutions (Table 7). The size of the communities was highly variable. The communities consisted of 2–139 institutions located in 1–26 countries. The largest community was headed by Philadelphia and networked mainly but not exclusively with North American partners. Only very few institutions in the United States did not cooperate within the CM 04 network. In Europe six communities were identified which were led by a European institution. The largest European network was truly international with 123 institutions from 26 countries, all other appeared more country or language oriented (CM 01 Italian, CM 02 German, CM 06 Spanish, CM 07 Polish, CM 11 French). The language character was also evident for the communities CM 05 (Japanese), CM 08 (Indian), CM 09 (Korean), CM 10 (Australian), CM 12 (Israel), CM 13 (Taiwan). Satellite institutions were excluded by definition from the community analysis.

**Table 7:** Identified networking communities.

community	#institutions	#countries	publications	citations	h-index	h_m_-index	mean weight	mean degree	mean closeness	mean betweenness
CM 01	45	6	3,064	85,576	116	4.01	8.171	40.622	0.396	0.003
CM 02	77	13	4,866	132,921	135	3.84	8.086	36.390	0.396	0.003
CM 03	123	26	6,963	191,143	146	3.57	8.079	57.203	0.415	0.004
CM 04	139	17	10,996	417,632	221	4.47	9.214	42.806	0.397	0.003
CM 05	68	4	3,464	94,130	116	3.8	6.438	25.441	0.368	0.003
CM 06	17	2	833	19,314	61	3.64	1.873	27.294	0.366	0.001
CM 07	11	1	221	2,875	27	2.81	2.003	25.091	0.339	0.001
CM 08	3	1	80	1,021	17	2.71	3.593	6.667	0.282	0.003
CM 09	2	1	612	10,464	46	3.12	5.250	5.000	0.301	0.002
CM 10	24	7	1,045	24,627	73	3.96	4.263	19.000	0.367	0.003
CM 11	2	1	30	1,574	18	4.32	1.450	6.000	0.335	0.000
CM 12	3	2	105	4,004	38	5.4	0.485	9.333	0.348	0.000
CM 13	6	1	423	7,399	38	3.01	6.844	5.667	0.271	0.003
sum	520	42	32,702	992,680						

The 520 institutions were separated into 13 communities using the Louvain method, the other 33 institutions were singletons and not identified as part of a larger network. Each institution, country, publication and citation counted only once within one community.

The h*-*index correlated well with the mean degree (R^2^ = 0.677), the mean weight (R^2^ = 0.644), the mean closeness (R^2^ = 0.601), and the mean betweenness (R^2^ = 0.401). Multivariably independent was only the mean weight variable (Table 1). The h_m_-index showed a weak association with the mean closeness (R^2^ = 0.237), while the mean weight (R^2^ = 0.013), degree (R^2^ = 0.017), and betweenness centralities (R^2^ = 0.112) were not correlated. The hazard ratios had very wide 95% confidence interval for all multivariable analyses of communities.

## Discussion

Long-term active presence on ANR congresses was strongly correlated with the h-index as an accepted measure of scientific productivity (number of original publications) and impact (number of citations).

Data on use of attending scientific conferences are sparse. The participation in such congresses precede the later published papers by years (in pediatric oncology estimated 4–5 years). Not all the abstracts will finally present as papers and not all authors of abstracts appear as authors of the related papers. Thus, the community of the congresses is wider and not congruent with the scientific community present in publications.

### 1. Strength and limitations of the study

The strengths of this study are the unchanged well-defined topic (neuroblastoma) and the long time period (4 decades). The continuing increase of abstracts, authors, institutions, countries, and the establishment of communities reflect a successful development of a scientific network. The neuroblastoma network may serve as a model to explore the structure and evolution of a scientific community. The names of identified top authors and institutions with high values were generally well-known while those with intermediate and low figures were much less. Thus, the objective data supported the subjective impression. Differences in citation behavior by the fields as discussed in the Leiden manifesto [13] -in this study related to basic, translational and clinical types of research- were not seen. This very likely results from the close collaborations e.g. between basic scientists and clinicians awarding mutually the contributions of each other: molecular data were obtained from the tissue of patients and woud be much less useful without the clinical background. Newly detected molecular markers were quickly into the clinics for risk estimation [1] and therapy e.g. targeting the molecule ALK with the drug crizotinib [14].

One important limitation of the study is the use of the comprehensive set of data over the complete observation period preferring the older, well-established persons and institutions and underestimating ‘rising stars’ and developments of shorter duration [13, 15, 16] This limitation applies not only to authors but also to institutions. For instance, institutions that started as satellites and fused later to the central network were not depicted as satellites.

### 2. The correlation of networking at congresses and publication success

The characteristic weight (fractionalized counting of authorship) [6] had a remarkable association with the h*-*index for authors and was one of the five independent factors in the multivariable model. This indicates that very active participants at the congress can often publish their works with high impact later on. The correlation for the h- and l-index, albeit lower than for weight, shows that authors with a high h-index can be found amongst a highly collaborative network. The work of Schubert et al. [11] attributed this correlation to the organic evolution of an organized network and partly to practices such as self- and cross-citation. Similar to [11], who investigated 36 journals in the field of Dentistry & Oral.

Medicine, we could also see a high correlation between the l-index and degree (“Fig 5F”, R^2^ = 0.817). This agrees to their observation that, as such a network grows, degree and l-index evolve in parallel, and that the l-index is a useful measure for centrality for author networks.

Another study [17], extended the concept of degree and l-index for weighted co-authorship networks, where edges are weighted by the number of common publications. In their investigation of authors and publications in the field of information science over two years, the incorporation of weights into centrality measures led to better correlation with h-index compared to unweighted centralities like degree and l-index. However, their correlations were generally lower than in our case (Spearman correlations Degree vs. H-index: 0,362873535, Weight vs. H-index: 0,421916616, l-Index vs. H-index: 0,34343135), possibly due to a sparser network due to the shorter timeframe (2 years vs. 41 year in the neuroblastoma network).

The h*-*index of institutions (n = 553) was best correlated with degree centrality followed by weight. For countries (n = 53), weight had the highest correlation with the h*-*index and remained as one of the two factors (together with degree) in the multivariable model. However, under no condition our correlations reached R^2^ = 0.99 as reported for the prediction of the future impact of researchers having 20–36 years of experience using h-index itself [15]. Predictions for researchers with one year experience were not precise in that h-index model (R^2^ = 0.60 for 1 year, R^2^ = 0.33 for 5 years).

The Molinari modification of the h-index correcting for the size dependency of an institution or of a country identified indeed largely different lists. The overlap between h-index and h_m_-index of the top 20 institutions was 45% (9/20) and of the top 10 countries 20% (2/10). The correlations between the networking activities of institutions at the congresses and the h_m_-index were weak. For the countries, degree centrality (R^2^ = 0.424; multivariable log rank p-value 0.007) showed the only relevant association. The citation rate of institutions and countries was highly correlated to the h_m_-index and thus showed similar and mostly even weaker associations to network centralities. The authors therefore conclude that the h_m_-index and citation rate were less useful for the analysis of this particular network. This may not be surprising, as the size of an institution, i.e. the number of researchers working there, directly affects the possibility and probability of widespread collaborations, e.g. by the acquisition of researchers from other institutions or the participation in international projects (R^2^ = 0.638 for size vs. degree). However, this effect can only be partly transferred to countries, possibly due to the smaller network (n = 53, R^2^ = 0.209 for size vs. degree).

### 3. Ranking lists

Ranking lists by h*-*index are considered to be helpful in identifying most relevant figures by quantitative measures. Regarding authors, the top 20 occupied similar good ranks in all five measures. The summarized ranks (Table 2) showed a relatively limited range (30–863; median 89.5; potential range 5–42,295). Thus, the study identified prominent authors already at congresses with sufficient reliability using degree centrality and/or weight. Most of the top authors were also in the top ranks of one of the centralities. This implies that authors with a higher publication rate and success are also part of a large and highly connected network within the congress. The data and the subjective impression were in good agreement.

Regarding institutions, the top 20 of the h-index list (Table 3) were all well-known. In particular the top five (Philadelphia as the founding institution of the congresses, Los Angeles, London, Bethesda, San Francisco) are recognized as strong and long-lasting institutions. The summarized networking ranks (range 5–1,200; median 110.5; potential range 4–2,212) demonstrated relatively narrow range in the h-index list compared to the h_m_-index list (range 5–1,676; median 308.5; potential range 4–2,212). By correlation, the best h-index predictors were weight and/or degree. Thus, the institutions showed a similar impact on the local interaction on the publication success. In contrast, the top 20 institutions of the h_m_-index list were less known (except the institutions Los Angeles, New York 1, and Philadelphia).

For countries, the top 10 (Tables 5 and 6) had a rank sum range of 4–74 (median 21; potential range 4–212) in the h-index list. The rank sums for the h_m_-index list were higher in most cases (range 28–88; median 61.5; potential range 4–212). The high influence of betweenness (correlation betweenness and h-index R^2^ = 0.704), compared to authors and institutions, emphasizes the brokerage role of the countries, but was multivariably not significant (log rank p = 0.294). The obtained data were in good agreement with the subjective impression.

### 4. Satellites and Louvain communities

520 institutions from 42 countries convened in 13 collaborative communities, while 33 institutions from 22 countries remained as ‘satellites’ unconnected to the large central network.

The detection of 33 satellite institutions attending one to four ANR congresses and lasting up to 16 years was surprising. The vast majority was located in developing countries. The identification of labile structures like satellite groups may become key to apply for more support, e.g. in developing countries from governmental sources. Teams from India and Belarus used already the ‘satellite information’ to call for more support from their local authorities. Investigations on the dynamics regarding appearance of satellites and merging into the central network are not yet done.

For the communities, the preferred collaboration within one community did not exclude cooperation with authors from other communities. The impressive international spread of the network based on one diagnosis of a pediatric cancer illustrates the large geographic extension. Closer views demonstrated the almost complete coverage of North America by community CM 04 and the more diverse communities of Europe (CM 01, 02, 03, 06, 07). The same language and geographical closeness might have been the starting points for scientific collaboration. With time far distant institutions were included into each of those communities. While research is shifting between teams, the time course of community building will be worthwhile to investigate. Larger communities are on average older, but the stability is based on a continuous change of their members [18]. The identified communities are in good agreement with the subjective impression of the authors regarding the preferences of collaborations between the groups. Although average degree, closeness, and betweenness centralities of the 13 communities (Table 7) and the compiled community h*-*index showed a correlation, the practical relevance of those measures appears of limited value in our case.

### 5. Other studies

The number of scientometric studies comparing the presence of scientists on conferences with the output of research papers is limited. Asnafi and coworkers [19] reported 137 papers of the 10 top authors who presented their work at a the scientometric conferences between 2003 and 2015. The network was mainly Iran-country based and demonstrated higher values as well for degree as for closeness centrality in the top ranks. Another study [20] investigated Web of Science publications from 2000 to 2013 and identified cancer research as the field with the highest levels of centralities (degree, closeness, betweenness, Eigenvector) in comparison to occupational health, gastrointestinal diseases, and internal medicine.

### 6. Implications of the study

The implications of the study are threefold.

- The study describes the development of a large scientific network built over 42 years. At the 18 congresses held, the number of papers, authors, institutions, and countries increased continuously in this field but not exponentially as in other fields [16]. The community of the congresses is wider and not congruent with the scientific community present in publications. A significant proportion of authors, and even institutions and countries presented at the ANR congresses only once (58% of author, 26% of institutions, 13% of countries). Scientists who participated not more than twice in congresses were irrelevant in the scientific literature related to the topic of the congresses. This demonstrates that it is of use to go to the congresses, but the active participation must continue. In contrast, whatever the criterium was (weight, centralities), the ranking lists sorted by the h*-*index displayed a remarkable congruity at top ranks in particular for authors. 85% of top 20 authors were among the top 20 in all lists (20 top institutions 40% congruity, 10 top countries 60%). Hosting an ANR congress had a (reversible) impact on the number abstracts. A surprising feature was the detection of satellite institutions without links to the central network up to 16 years.
- The geographical distribution of scientists has been central in North America, Europe, Japan, and Australia. Preferred scientific collaborations were identified using the Louvain algorithm for communities showing the far-reaching links.
- Frequent presence at congresses was correlated with high numbers of publications and citations. The authors were surprised detecting that continued (weighted) contributions to congresses predicted later scientific success (number of papers and number of citations evaluated as h-index). The h*-*index as measure of recognition by the scientific community appeared more useful than the h_m_-index. Degree, closeness, and betweenness centralities at congresses were variably useful to predict h*-*index for authors, institutions, countries, and communities, while weight (fractionalized counting) [6] was helpful in all instances. Therefore, the authors suggest including weight as a useful variable for a first estimation to potential future publication success.

In conclusion, this study describes the characteristics of a scientific network growing worldwide over more than 40 years and provides new insights into the function of congresses for scientific networking and publication success. The emergence of new scientific structures and knowledge is very complex and so far not much is known about. We hope that our contribution adds a little piece to the puzzle.

## Supporting information

S1 File PONE-D-18-25965R1_FAR supp._inf._file_1.xlsx. (XLSX)

S2 File PONE-D-18-25965R1_FAR supp._inf._file_2.xlsx. (XLSX)

S3 File PONE-D-18-25965R1_FAR supp._inf._file_3.xlsx. (XLSX)

S4 File PONE-D-18-25965R1_FAR supp._inf._file_4.xlsx. (XLSX)
