# Correction: Are network growth and the contributions to congresses associated with publication success? A pediatric oncology model

**DOI:** 10.1371/journal.pone.0212536

**Published:** 2019-02-13

**Authors:** The PLOS ONE Staff

## Notice of republication

An incorrect version of S1 File was published in error. The publisher apologizes for this error. This article was republished on February 6, 2019 to correct for this error. Please download this article again to view the correct version.
